# A Network Comprised of miR-15b and miR-29a Is Involved in Vascular Endothelial Growth Factor Pathway Regulation in Thymus Adipose Tissue from Elderly Ischemic Cardiomyopathy Subjects

**DOI:** 10.3390/ijms241914456

**Published:** 2023-09-22

**Authors:** Adriana Mariel Gentile, Said Lhamyani, María Mengual Mesa, Francisco Javier Pavón-Morón, John R. Pearson, Julián Salas, Mercedes Clemente-Postigo, Lucía Pérez Costillas, Gabriel Olveira Fuster, Rajaa El Bekay Rizky

**Affiliations:** 1 Instituto de Investigación Biomédica de Málaga y Plataforma en Nanomedicina-IBIMA Plataforma BIONAND, 29580 Malaga, Spain; biogentile@gmail.com (A.M.G.); saidlhamyani@gmail.com (S.L.); maria.mengual.mesa@gmail.com (M.M.M.); javier.pavon@ibima.eu (F.J.P.-M.); mer.cp@hotmail.com (M.C.-P.); gabolvfus@uma.es (G.O.F.); 2 Clinical Unit of Endocrinology and Nutrition, University Regional Hospital of Malaga, 29009 Malaga, Spain; 3 Andalucía Tech, Faculty of Health Sciences, and Department of Systems and Automation Engineering, School of Industrial Engineering, Universidad de Málaga, Teatinos Campus, s/n, 29071 Málaga, Spain; 4 Clinical Unit of the Cardiology Area, University Hospital Virgen de la Victoria, 29009 Málaga, Spain; 5 Spain Biomedical Research Networking Center on Cardiovascular Diseases (CIBERCV), Health Institute III, 28029 Madrid, Spain; 6 Biomedicine Institute of Seville (IBiS), 41013 Seville, Spain; johnrpearson@gmail.com; 7 Department of Cardiovascular Surgery, University Regional Hospital of Malaga, 29009 Malaga, Spain; jsalas@telefonica.net; 8 Maimónides Biomedical Research Institute of Córdoba (IMIBIC), Reina Sofia University Hospital, Department of Cell Biology, Physiology and Immunology, University of Córdoba, 14004 Córdoba, Spain; 9 Spanish Biomedical Research Center in Physiopathology of Obesity and Nutrition (CIBERObn), Instituto de Salud Carlos III, 28029 Madrid, Spain; 10 Research Unit, International Institute for Innovation and Care in Neurodevelopment and Language, Department of Psychiatry and Physiotherapy, Faculty of Medicine, University of Malaga, 29010 Malaga, Spain; lpcostillas@gmail.com; 11 Biomedical Research Networking Center on Diabetes and Associated Metabolic Diseases (CIBERDEM), Carlos III Health Institute, 28029 Madrid, Spain

**Keywords:** aging, angiogenesis, elderly, ischemia, microRNA, myocardial infarction, thymus adipose tissue, subcutaneous adipose tissue

## Abstract

As the human thymus ages, it undergoes a transformation into adipose tissue known as TAT. Interestingly, in previous research, we observed elevated levels of vascular endothelial growth factor A (VEGFA) in TAT from patients with ischemic cardiomyopathy (IC), particularly in those over 70 years old. Moreover, in contrast to subcutaneous adipose tissue (SAT), TAT in elderly individuals exhibits enhanced angiogenic properties and the ability to stimulate tube formation. This makes TAT a promising candidate for angiogenic therapies and the regeneration of ischemic tissues following coronary surgery. MicroRNAs (miRNAs) have emerged as attractive therapeutic targets, especially those that regulate angiogenic processes. The study’s purpose is to determine the miRNA network associated with both the VEGFA pathway regulation and the enrichment of age-linked angiogenesis in the TAT. RT-PCR was used to analyze angiogenic miRNAs and the expression levels of their predicted target genes in both TAT and SAT from elderly and middle-aged patients treated with coronary artery bypass graft surgery. miRTargetLink Human was used to search for miRNAs and their target genes. PANTHER was used to annotate the biological processes of the predicted targets. The expression of miR-15b-5p and miR-29a-3p was significantly upregulated in the TAT of elderly compared with middle-aged patients. Interestingly, *VEGFA* and other angiogenic targets were significantly upregulated in the TAT of elderly patients. Specifically: *JAG1*, *PDGFC*, *VEGFA*, *FGF2*, *KDR*, *NOTCH2*, *FOS*, *PDGFRA*, *PDGFRB*, and *RHOB* were upregulated, while *PIK3CG* and *WNT7A* were downregulated. Our results provide strong evidence of a miRNA/mRNA interaction network linked with age-associated TAT angiogenic enrichment in patients with IC.

## 1. Introduction

Acute myocardial infarction (MI) produces massive injury to the coronary microcirculation, which leads to ischemic cardiomyopathy and generally results in myocardial necrosis. Coronary myocardial perfusion is typically required to avoid heart failure, and tissue repair after MI involves a mechanical angiogenic response [1]. Although the incidence of mechanical complications remains low, the associated mortality rate is high, especially among elderly patients [2]. Therefore, more effective angiogenic therapies are vital for this group of patients.

Currently, adipose tissue is the most suitable source of angiogenic factors available [3], and it is an attractive candidate for tissue engineering and neovascularization [4]. Although the available data have shown that aging seriously impairs the angiogenic properties of adipose tissue [5,6], it is recognized as the most suitable source of angiogenic factors and progenitor cells to promote neovascularization in ischemic injuries because of its ease of access [6].

On the other hand, the aging of the thymus is known to be a fast process in all mammals. In humans, thymic senescence begins at puberty and by 50 years of age, 80% of the thymic epithelium is replaced by adipose tissue [7]. The thymus adipose tissue (TAT) is partially discarded during the aortic cannulation procedures performed as part of a cardiopulmonary bypass, which makes TAT a readily available source of fat and adipokines [8]. Previously, we have shown that adult TAT from patients with ischemic cardiomyopathy produces a variety of angiogenic factors and induces proliferation and migration of human umbilical cord endothelial cells, which suggests its angiogenic potential [9]. In contrast to other adipose tissues (e.g., the subcutaneous adipose tissue [SAT]), the angiogenic characteristics of TAT are significantly increased with aging [8]. These data suggest that the TAT could be an ideal source of angiogenic and endothelial factors for elderly patients with ischemic cardiomyopathy. These properties are likely to be controlled by vascular endothelial growth factor A (VEGFA) because it is the angiogenic factor most highly upregulated in adult TAT as compared with SAT [8]. VEGFA is the single most important regulator of blood vessel formation; it is essential for embryonic angiogenesis and vasculogenesis, and it is a key mediator of neovascularization [10].

MicroRNAs (miRNAs) are non-coding RNAs that regulate gene expression and have emerged as important factors in health and disease [11]. The expression of miRNAs varies during all stages of myocardial ischemia reperfusion and subsequent ischemia-reperfusion injury [12]. These changes suggest that miRNAs may have a functional role in the ischemic processes and thus make for attractive therapeutic targets given the upregulation of angiogenic VEGFA in the TAT and the potential involvement of miRNA regulation in ischemic cardiomyopathy.

The primary objective of this study is to identify the specific miRNAs that exhibit differential expression in the TAT. Additionally, we aim to investigate the potential regulatory networks of these miRNAs, particularly their involvement in modulating the VEGFA pathway and contributing to age-related angiogenic processes within the TAT.

## 2. Results

### 2.1. Clinical and Biological Variables of Both Patient Groups

The study was performed in two groups of patients who had CABG surgery: (1) the elderly group (i.e., ≥70 years of age) with 10 patients and (2) the middle-aged group (i.e., 45 to 65 years of age) with 8 patients. These patients had a mean number of 3.1 grafts per patient.

Table 1 shows that there were no significant differences in all anthropometric and biochemical variables between the two groups, except for age, as expected (mean age of 56.6 and 74.0 years, respectively; *p* < 0.001).

### 2.2. In Silico Identification of miRNAs Predicted to Regulate VEGFA and TargetScore Calculation of VEGFA miRNA Binding Sites

**Figure 1:**
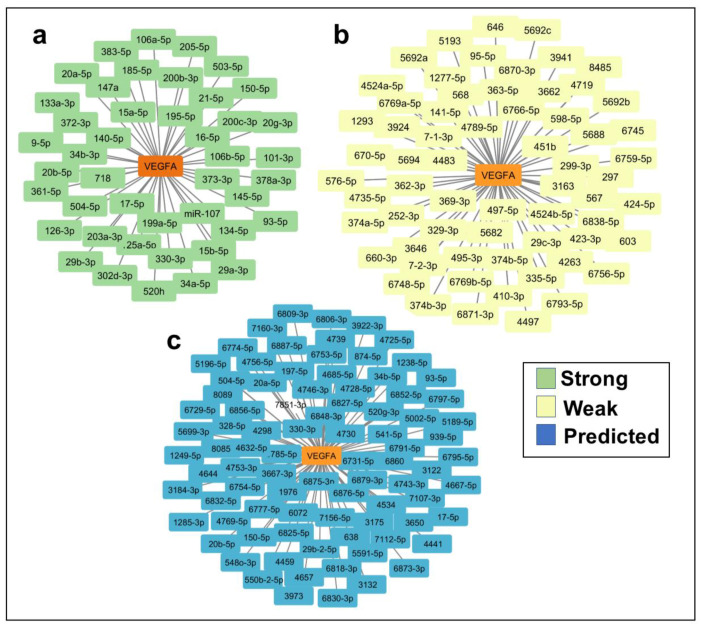
In silico analysis of miRNAs predicted to regulate VEGF-A. (**a**) MiRTargetLink Human was used to identify 44 miRNAs as strong potential VEGF-A regulators. (**b**) MiRTargetLink Human was used to identify 64 miRNAs as weak probability VEGF-A regulators. (**c**) MiRTargetLink Human was used to identify 87 miRNAs as probability VEGF-A regulators.

To examine the potential involvement of miRNAs in the regulation of VEGFA, we performed an in silico analysis of the VEGFA gene sequence using miRTargetLink Human [13]. This search identified 195 miRNAs as potential regulators of VEGFA (Figure 1a–c, and Table S1). Of these miRNAs, 44 were evaluated as having a high probability of involvement in VEGFA regulation (Figure 1a), 64 were evaluated as having a low probability (Figure 1b), and 87 were evaluated as predicted target genes (Figure 1c). Since miRNA–mRNA binding sites are not equally effective, we used the TargetScan tool [14] to calculate TargetScore values for each miRNA. This allowed us to pinpoint the five miRNAs (miR-15b-5p, miR-16-5p, miR-29a-3p, miR-29b-3p, and miR-195-5p) predicted to have the highest binding effectiveness. Consequently, these miRNAs are the most likely candidates for regulating VEGFA (Table S2).

Previously, we have shown that miR-21-5p regulates VEGFA in adipose tissue [15]. Although miR-21-5p does not have a high-probability binding site in VEGFA, we believe that it may act indirectly, and therefore it was also included in subsequent analyses.

In our previous study, we established miR-21-5p’s regulatory role in *VEGFA* within adipose tissue [15]. Despite the absence of a high-probability binding site for miR-21-5p in *VEGFA*, we believe it may exert an indirect influence. For this reason, we believe it would be highly valuable to include it in the analysis.

**Figure 2:**
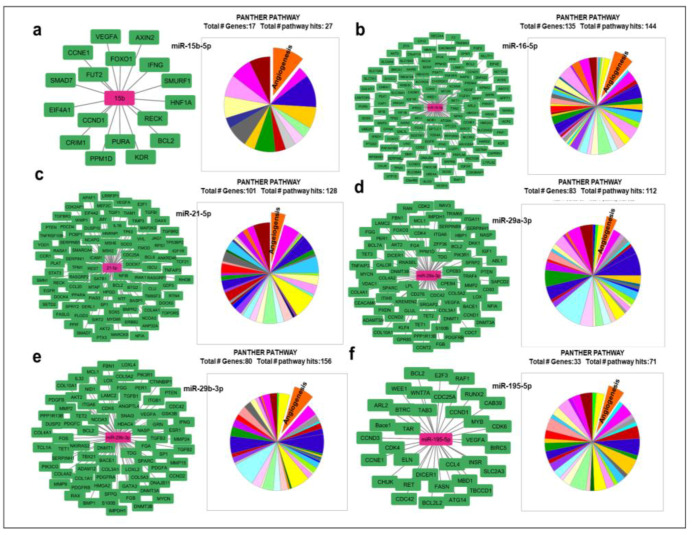
In silico analysis of angiogenic target genes. Target genes (green nodes) that could be regulated by miR-15b-5p; miR-16-5p; miR-29a-3p; miR-29b-3p; miR-195-5p; and miR-21-5p (fuchsia nodes) using MiRTargetLink Human to identify all potential mRNA target genes and the PANTHER database to identify those with a role in angiogenic processes. (**a**) Seventeen mRNAs were identified as potential miR-15b-5p target genes, of which two were angiogenesis related. (**b**) One hundred thirty-five mRNAs were identified as potential miR-16-5p target genes, of which nine were angiogenesis related. (**c**) Eighty-three mRNAs were identified as potential miR-29a-3p target genes, of which three were angiogenesis related. (**d**) Eighty mRNAs were identified as potential miR-29b-3p target genes, of which 10 were angiogenesis related. (**e**) Thirty-three mRNAs were identified as potential miR-195-5p target genes, of which three were angiogenesis related. (**f**) One hundred one mRNAs were identified as potential miR-21-5p target genes, of which five were angiogenesis related.

In order to find out whether these miRNAs might also regulate other angiogenic genes, we used miRTargetLinks to identify putative target genes of the six selected miRNAs (miR-15b-5p; miR-16-5p; miR-29a-3p; miR-29b-3p; miR-195-5p; and miR-21-5p). We identified several target genes as having a high probability of being regulated (Figure 2a–f), of which 25 were related to angiogenesis regulation (Figure 2a–f and Table S3). 

### 2.3. Expression Profile of miR-15b-5p, miR-16-5p, miR-29a-3p, miR-29b-3p, miR-195-5p, and miR-21-5p in Human TAT and SAT

Since TAT displays an age-associated rise in VEGFA expression [8], we analyzed the expression levels of miRNAs that may be implicated in regulating this angiogenic factor in both TAT and SAT samples collected from elderly and middle-aged patient groups using RT-qPCR.

**Figure 3:**
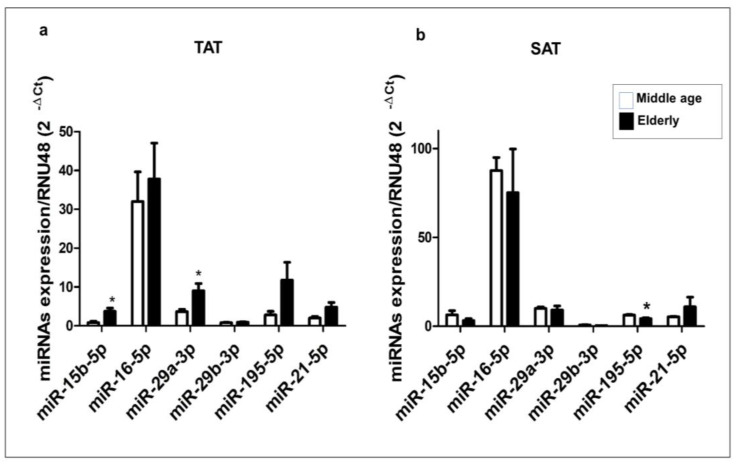
Expression profiles of miR-15b-5p, miR-16-5p, miR-29a-3p, miR-29b-3p, miR-195-5p, and miR-21-5p in human TAT and SAT. miRNA expression levels were measured by RT-qPCR, and the relative quantification of expression levels was carried out using the comparative threshold cycle (Ct) method. The hsa-RNU48 was used as an endogenous control. Data are expressed as the mean ± SEM and assessed using the Mann–Whitney U test to compare the elderly and middle-aged patient groups. (**a**) miRNAs were extracted from TAT (*n* = 18). (**b**) miRNAs were extracted from SAT (*n* = 18). * *p* ≤ 0.05.

Within the TAT, we observed a notable increase in the expression levels of miR-15b-5p and miR-29a-3p in the elderly group compared to the middle-aged group (*p* < 0.05). However, no significant differences were found in the expression of miR-16-5p, miR-21-5p, miR-195-5p, and miR-29b-3p between the two patient groups (Figure 3a). 

The SAT displayed a notably distinct miRNA expression profile compared to the TAT. Specifically, the expression level of miR-195-5p was significantly lower in the elderly group than in the middle-aged group (*p* < 0.05), while no significant differences were observed in the expression of the remaining miRNAs (Figure 3b). This highlights a potential association between TAT and age-specific expression of four miRNAs in relation to VEGFA, including three miRNAs that we anticipate have robust interactions with VEGFA mRNA.

### 2.4. mRNA Expression Profile of Putative Target Genes in Human TAT 

**Figure 4:**
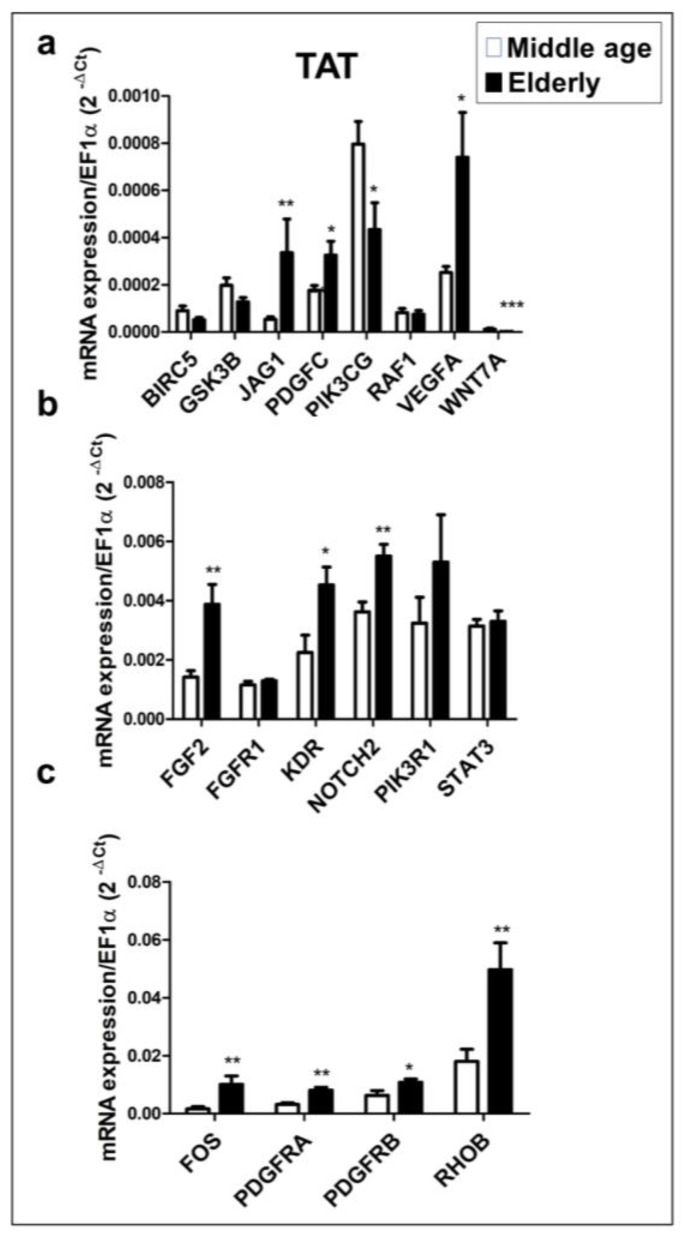
Expression profiles of 18 potential angiogenic target genes of miR-15b-5p, miR-16-5p, miR-29a-3p, miR-29b-3p, miR-195-5p, and miR-21-5p. (**a**) mRNA expression level of BIRC5, GSK3B, JAG1, PDGFC, PIK3CG, RAF1, VEGFA and WNT7A). (**b**) mRNA expression level of FGF2, FGFR1, KDR, NOTCH2, PIK3R1 and STAT3. (**c**) mRNA expression level of FOS, PDGFRA, PDGFRB and RHOB. The mRNAs were extracted from TAT (*n* = 18). mRNAs expression levels were measured by RT-qPCR, and relative quantification of the expression levels was carried out with the comparative threshold cycle (Ct) method. EF1α was used as an endogenous control. Data are expressed as the mean ± SEM and assessed using the Mann–Whitney U test to compare the elderly and middle-aged patient groups. * *p* ≤ 0.05; ** *p* ≤ 0.01; *** *p* ≤ 0.001.

Because we previously found that the six candidate miRNAs are predicted to interact with several angiogenic genes in addition to VEGFA, we performed a RT-qPCR to compare the expression levels of the predicted target genes in the TAT obtained from the elderly and middle-aged patient groups. Our analysis found that 18 of the 25 predicted target genes were expressed in the TAT, with 11 target genes showing statistically significant differences between the two patient groups (Figure 4). The expression levels of JAG1, PDGFC, VEGFA (Figure 4a), FGF2, KDR, NOTCH2 (Figure 4b), FOS, PDGFRA, PDGFRB, and RHOB (Figure 4c) were significantly higher in the elderly group than in the middle-aged group (*p* < 0.05 and *p* < 0.01). Conversely, the expression levels of PIK3CG and WNT7A (Figure 4a) were significantly lower in the elderly group than in the middle-aged group. We found no significant differences in the expression levels of BIRC5, GSK3B, RAF1 (Figure 4a), FGFR1, PIK3R1, and STAT3 (Figure 4b) between the two patient groups.

### 2.5. Interaction Network Model between VEGFA, the miRNAs, and Target Genes in Human TAT of Elderly Patients 

**Figure 5:**
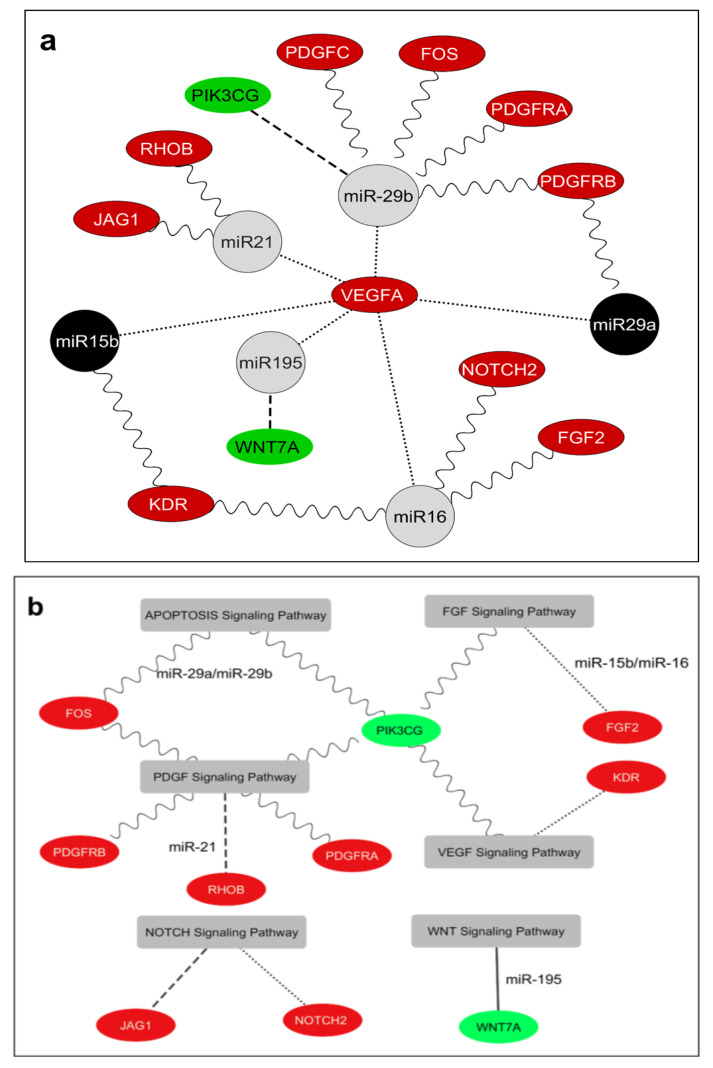
(**a**) Interaction network between miR-15b-5p, miR-16-5p, miR-29a-3p, miR-29b-3p, miR-195-5p, and miR-21-5p and their 12 angiogenic target genes in human TAT. Cytoscape v.3.2.1 software (NIGMS. 45 Center Drive, MSC 6200, Bethesda, Maryland 2089-6200) was used to draw the interaction network between miRNAs and their 12 angiogenic target genes differently expressed and considering the comparative results of expression levels of these miRNA/mRNAs between elderly and middle-aged patients. Grey nodes show the miRNAs that have similar expression levels in both elderly and middle-aged patients. Black nodes show the miRNAs that are upregulated in elderly patients compared with middle-aged patients; green nodes show the mRNAs that are downregulated in elderly patients compared with middle-aged patients; and red nodes show the mRNAs that are upregulated in elderly versus middle aged patients. Lines show potential interaction, direct or indirect, between miRNAs and their target genes; dots indicate potential upregulation of VEGFA; dashes indicate downregulated target genes; sine waves indicate potentially upregulated target genes. (**b**) Cytoscape was used to draw the putative interaction network among miRNAs, and 12 target genes and predicted pathways. Green nodes show the mRNAs that are downregulated in elderly patients compared with middle-aged patients in TAT, and red nodes show the mRNAs that are upregulated in elderly versus middle-aged patients in TAT. Grey nodes show the pathways where the genes are involved. Lines show potential participation of target genes in different signaling pathways. Sine waves indicate potential regulation by miR-29a/miR-29b cluster; dots indicate potential regulation by miR-15b/miR-16 cluster; dashes indicate potential regulation by miR-21; and solid lines indicate potential regulation by miR-195. Here, only predicted interactions are shown.

Collectively, our bioinformatics-derived data strongly indicate the presence of a complex regulatory network in the TAT. This network involves age-related differential expression of miRNAs, namely miR-15b-5p, miR-16-5p, miR-29a-3p, miR-29b-3p, miR-195-5p, and miR-21-5p, as well as a group of 12 angiogenesis-promoting genes, including *PDGFC*, *PIK3CG*, *VEGFA*, *WNT7A*, *FGF2*, *JAG1*, *KDR*, *NOTCH2*, *FOS*, *PDGFRA*, *PDGFRB*, and *RHOB* (Figure 5a). Additionally, through predictive bioinformatics analysis, we were able to elucidate the specific angiogenic pathways they are involved in (Figure S1 and Table S4). Indeed, a bivariate correlation analysis among miRNAs revealed significant positive associations: miR-21-5p and miR-195-5p (R = 0.938; *p* = 0.0001), miR-15b-5p and miR-16-5p (R = 0.890; *p* = 0.001), and miR-16-5p and miR-29b-3p (R = 0.735; *p* = 0.16). On the other hand, miRNA–mRNA bivariate correlations demonstrated positive associations: miR-29b-3p and *KDR* (R = 0.694; *p* = 0.026), miR-15b-5p and *NOTCH2* (R = 0.613; *p* = 0.05), and a negative association between miR-29a-3p and *RHOB* (R = −0.641; *p* = 0.046). 

Additionally, bivariate mRNA–mRNA correlations unveiled a negative correlation between *PIK3CG* and *KDR* (R = −0.683; *p* = 0.029), *PIK3CG* and *FGF2* (R = −0.618; *p* = 0.05), *PIK3CG* and *NOTCH2* (R = −0.700; *p* = 0.024), and *PIK3CG* and *PDGFRA* (R = −0.877; *p* = 0.001) and a positive correlation between *VEGFA* and *KDR* (R = 0.741; *p* = 0.014), KDR and *PDGFRB* (R = 0.715; *p* = 0.02), *FGF2* and *PDGFRA* (R = 0.685; *p* = 0.029), *FOS* and *PDGFC* (R = 0.782; *p* = 0.007), *NOTCH2* and *PDGFRA* (R = 0.766; *p* = 0.01), *NOTCH2* and *PDGFRB* (R = 0.625; *p* = 0.05), and *PDGFC* and *PDGFRA* (R = 0.735; *p* = 0.015).

## 3. Discussion

Previously, we presented evidence that the TAT is an attractive new source of adipose tissue and angiogenic factors highlighting its angiogenic properties improve in old age, while these characteristics are impaired in the SAT [8,16]. Moreover, we also showed that *VEGFA* is the most upregulated angiogenic factor in the TAT in comparison with the SAT [8]. The miRNAs are recognized to play a pivotal role in inducing angiogenesis for the treatment of ischemic diseases [17], and alterations in their expression during aging contributes to an age-dependent decline in vascular function and angiogenesis [18]. Thus, we considered it necessary to extend our previous studies by analyzing the potential role of miRNAs in the regulation of angiogenic properties in adipose tissue, especially the TAT. 

Our findings suggest that the upregulation of the VEGFA pathway may be linked to changes in the expression of specific miRNAs, specifically miR-15b and miR-29a.

The miR-15 family comprises six highly conserved members: miR-15a, miR-15b, miR-16-1, miR-16-2, miR-195, and miR-497 [19]. Among these, three are expressed in the TAT of elderly patients, namely miR-15b, miR-16, and miR-195. It is important to note that members of a miRNA cluster or family exhibit different expression levels and co-expression patterns, suggesting potential miRNA–miRNA interactions both within and between clusters or families. Additionally, the expression of each miRNA within the same cluster or family is dependent on the expression of other members of the cluster [20]. 

In this context, it has been reported that the miR-15b/miR-16 cluster contributes to the upregulation of *VEGFA* [20,21,22]. Therefore, based on our results, the overexpression of miR-15b and co-expression of miR-16 may be associated with an upregulation of *VEGFA*. Our findings also demonstrate a positive correlation in expression levels between miR-16 and miR-29b (R = 0.735; *p* = 0.016) and between miR-195 and miR-21 (R = 0.938; *p* = 0.0001). This suggests that their co-expression with members of the miR-15 or miR-29 family may promote the expression of various key genes involved in angiogenesis. Although miRNAs from the same cluster may not follow the same expression pattern, as seen with miR-15b and miR-16 or miR-29a and miR-29b, where one shows an upregulation and the other does not alter its gene expression, it cannot be ruled out that they are not involved in the regulation of the VEGFA pathway. The mere fact that they are expressed could contribute as significant members of their cluster in the regulation of *VEGF*, particularly in the case of the TAT. 

Furthermore, it is known that the Wnt/β-catenin signaling pathway plays a significant role in the angiogenic activity of endothelial cells (ECs) [23]. In relation to this, a study highlights three highly conserved members of the miR-15 family: miR-15a, miR-15b, and miR-195, which could serve as critical regulators of *WNT7A*. Among them, miR-15b demonstrated an inverse correlation with *WNT7A* [19]. This phenomenon may also be occurring in the TAT of elderly patients, as our results indicate an inverse expression relationship between miR-15b and *WNT7A*.

Moreover, it is widely recognized that neovascularization involves the activation, proliferation, and migration of mature endothelial cells that extend the pre-existing vascular network (angiogenesis). Additionally, it is known that endothelial progenitor cells (EPCs) can promote neovascularization through the paracrine secretion of angiogenic factors and cytokines [24]. Indeed, a study demonstrates the dysregulation of miRNAs in these cells, including members of the miR-29 family, which may play a pivotal role in modulating angiogenesis and the function of EPCs. This includes VEGF signaling, extracellular matrix remodeling, PI3K/AKT/MAPK signaling, the transforming growth factor-beta (*TGFβ*) pathway, *p53*, and cell cycle progression [24]. In this context, we have shown a differential expression of miR-29a and *VEGFA*, which may align with this observation.

In our previous study, we highlighted that KDR (VEGF-R2) exhibits higher expression levels in TAT compared to SAT, with thymic expression being particularly elevated in elderly patients compared to middle-aged patients [8]. In the current investigation, we once again demonstrate an upregulation of *KDR*. Our findings are in line with research indicating that the primary pro-angiogenic signal arises from *VEGF-R2* activated by *VEGF* [25]. Furthermore, another study underscores a direct regulation of *KDR* by miR-15b, which aligns with our results [26].

Microvascular network growth and the angiogenic properties of the TAT in elderly patients with cardiomyopathy also seem to involve the upregulation of *PDGFC*, *FGF2*, *NOTCH2*, *FOS*, *JAG1*, and *PDGFRA* target genes. In fact, all these factors are mainly involved in angiogenesis, adipogenesis, and the *VEGF* and *WNT* signaling pathways [27,28,29,30,31,32,33,34,35]. This finding is outstanding, given that in other tissues there is an impaired angiogenesis that is associated with aging and is typically accompanied by decreased expression of these angiogenic factors [36].

PI 3-Kinase plays a pivotal role in neovascularization and angiogenesis, recognized as a key signaling molecule orchestrating various cellular functions such as growth, survival, and migration. This enzyme also influences the permeability of endothelial cells and the production of nitric oxide, as evidenced by studies [37,38]. The implication of this kinase in regulating the angiogenic response is complex and highly context dependent. It can be either overexpressed or inhibited depending on a range of factors, including the cell type involved, the specific microenvironment, and the presence of other regulators. The results from this study imply that the downregulation of PI3 kinase in the TAT of elderly subjects may serve as a crucial factor within the identified miRNA/mRNA network and potentially could play a crucial role in enhancing the angiogenic function that is specific to the TAT in these elderly individuals, as described in our previous study [8,9,16]. In support of our statement, the correlation analysis has revealed a negative connection between the expression levels of the angiogenic genes *KDR*, *FGF2*, and *PDGFRA* and *PIK3CG*.

Our results highlight an intriguing miRNA/mRNA network that may play a significant role in regulating the angiogenic functionality of the elderly TAT, as described in our previous studies [8,9,16]. In these previous studies, we found that TAT exhibits tube formation capacity, enhanced cellular production, vascular endothelial growth factor secretion levels, and the ability to promote endothelial cell survival [39]. Additionally, we have demonstrated elevated angiogenic effects of TAT compared to SAT in elderly individuals [8]. Therefore, our findings provide new insights into the potential upregulation mechanism of this angiogenic function in TAT, implicating a miRNA/mRNA network well known for its role in angiogenesis and the generation of new blood vessels. This network primarily involves a crucial factor, VEGF, known to be instrumental in inducing neovascularization. Both experimental and clinical evidence has underscored VEGF’s pivotal role in promoting neovascularization [40]. Furthermore, we highlight the involvement of *FGF2* in this network, which has been utilized in preclinical and clinical studies to stimulate the formation of new blood vessels in damaged or poorly vascularized tissues. It can be administered directly or through tissue engineering techniques [41,42]. Additionally, PDGF has been considered in angiogenic therapy for its potential to promote the proliferation and migration of endothelial cells, thus stimulating angiogenesis in tissues requiring it. Furthermore, PDGFD has been shown to enhance the angiogenic capacity of endothelial progenitor cells, including proliferation, migration, adhesion, and tube formation, thereby contributing to angiogenesis [43]. Moreover, given that miR29a and miR15b are integral components of this network, we believe that these miRNAs could play a significant role in promoting neovascularization and angiogenesis within elderly TAT. 

Therefore, conducting in vitro and in vivo studies with these miRNAs to elucidate their potential role in regulating the angiogenic functionality of TAT, as well as their possible contribution to the utilization of TAT in regenerative medicine and neovascularization, holds significant promise. We believe that a comprehensive examination of its involvement in regulating the enhancement of the angiogenic function of the elderly TAT could offer fresh and compelling insights in the field of investigating the neovascularization and microvascular network development potential of adipose tissue, presenting a notable challenge and opportunity for future therapeutic advancements in regenerative medicine [44].

## 4. Materials and Methods

### 4.1. Ethical Issues

The study was approved by the local ethics committee of Málaga Regional University Hospital (code number: PI07/00288) and performed in accordance with the Ethical Principles for Medical Research Involving Human Subjects adopted in the Declaration of Helsinki by the World Medical Association and the Regulation (EU) 2016/679 of the European Parliament and of the Council 27 April 2016 on the protection of natural persons with regard to the processing of personal data and on the free movement of such data (General Data Protection Regulation). The written informed consents were obtained from all participants by the Spanish Thoracic and Cardiovascular Surgery Society (SECTCV).

### 4.2. Participants and CABG Surgery

Eighteen patients who had coronary artery bypass graft (CABG) surgery with cardiopulmonary bypass as consequence of ischemic cardiomyopathy were recruited from the department of Cardiology at Hospital Regional Universitario de Málaga. CABG surgery is a procedure that uses the veins or arteries of the patients to bypass narrowed areas in order to restore blood flow to myocardium. Patients were divided into two groups according to aging: elderly and middle-aged groups.

All patients who were recruited were medically stable without severe ischemic injury. Patients had no infarction or prior infarction at least 6 months before surgery. None of them suffered from leg ischemic peripheral arteriopathy, which could potentially alter the expression of genes encoding angiogenic factors.

### 4.3. TAT and SAT Collection and Isolation of miRNA and mRNA

The tissue samples of SAT and TAT were removed at the beginning of the CABG practice and before the cardiac arrest. The SAT was obtained from the chest incision during the CABG. All tissue samples were immediately stored at −80 °C prior to subsequent procedures.

### 4.4. miRNA Extraction and RT-qPCR

miRNAs were isolated from adipose tissue according to the procedure described previously [13]. The mirVana™ miRNA Isolation Kit (Invitrogen, Thermo Fisher Scientific, Waltham, MA, USA) was used according to the manufacturer’s guidelines. miRNA concentration and purity were determined by NanoDrop1000 spectrophotometer (Thermo Fisher Scientific, Waltham, MA, USA) software ND-1000 v3.7.1. Complementary DNA (cDNA) was obtained using the TaqMan^®^ MicroRNA Reverse Transcription Kit (Applied Biosystems, Thermo Fisher Scientific, Waltham, MA, USA) (8 ng RNA/5 μL) and specific primers and probes for each miRNA were used (TaqMan^®^ MicroRNA Assay from Applied Biosystems, Thermo Fisher Scientific, Waltham, MA, USA): hsa-miR-15b-5p (assay ID 000390); hsa-miR-16-5p (assay ID 000391); hsa-miR-21-5p (assay ID 000397); hsa-miR-29a-3p (assay ID 002112); hsa-miR-29b-3p (assay ID 000413); hsa-miR-195-5p (assay ID 000494). Reverse transcription was performed as described previously [34], and miRNA expression levels were measured by Stratagene Mx3005p real-time polymerase chain-reaction system (RT-qPCR) (Agilent Technologies, Santa Clara, CA, USA). All samples were analyzed in duplicate, and the relative quantification of miRNA levels was performed using the comparative threshold cycle (Ct) method according to the manufacturer’s guidelines. hsa-RNU48 (TaqMan^®^ MicroRNA Assay ID 001006 from Applied Biosystems, Thermo Fisher Scientific, Waltham, MA, USA) was assessed using Bestkeeper© software to verify its usability as a reference gene (http://www.gene-quantification.de/bestkeeper.html) (accessed on 7 April 2021) and hsa-RNU48 was used as the endogenous control.

### 4.5. mRNA Isolation and RT-qPCR

The RNeasy^®^ Lipid Tissue Mini Kit (Qiagen,) was used to isolate total mRNA from adipose tissue and RNA concentration and purity were determined using a NanoDrop1000 spectrophotometer (Thermo Fischer Scientific, Waltham, MA, USA) software ND-1000 v3.7.1. cDNA synthesis was achieved using Transcriptor Reverse Transcriptase Kit (Roche Diagnostics, Barcelona, Spain) according to the manufacturer’s instructions and the RT-qPCR performed using Ultra-Fast SYBRGreen qPCR Master mix and assayed in a Mx3005p qPCR system (Agilent Technologies, Santa Clara, CA, USA).

ΔCt values for each PCR product were established using a threshold value. The reference gene was selected using Bestkeeper© software (http://www.gene-quantification.de/bestkeeper.html) (accessed on 4 November 2021). Primers used for RT-qPCR reactions are detailed in Table S5

miRTargetLink Human software [13] (https://ccb-web.cs.uni-saarland.de/mirtargetlink/) (accessed on 26 November 2020) was used to search for miRNAs that could be regulating *VEGFA* and to predict putative miRNA target genes. The TargetScan Human 7.2 tool [14] was used to calculate TargetScore, which measures conservation strength (site type: mer8 > 7mer-m8 > 7mer-A1 > 6mer), evolutionary conservation and targeting efficiency (context++ score percentile), and biological relevance (site conservation feature; P_CT_) as described in (34). The PANTHER Database (Protein ANalysis Through Evolutionary Relationship) Classification System (http://www.pantherdb.org/) (accessed on 26 November 2020) was applied to annotate the biological processes of target genes. Potential interactions between miRNAs, angiogenesis, and the expressed target genes and their pathways were visualized with Cytoscape v.3.2.1 software)(NIGMS: 45 Center Drive, MSC 6200, Bethesda, Rockville, MD, USA, 20892-6200. (http://www.cytoscape.org/) (accessed on 29 August 2023). 

### 4.6. Statistical Analysis

The results are expressed as the mean ± standard error of the mean (SEM). The Shapiro–Wilk test was used to assess the normal distribution of continuous variables, while variance heterogeneity was analyzed using the Levene’s test. Subsequently, data were analyzed using the Student’s *t*-test or Mann–Whitney U test. A bivariate correlation analysis was used to determine the association between miRNAs and their TGs. All statistical analyses were carried out with the statistical software package SPSS (version 22.0 SPSS; IBM, Chicago, IL, USA). We considered statistically significant *p* values equal to or less than 0.05 (*p* ≤ 0.05).

## 5. Conclusions

In summary, our findings strongly indicate the involvement of novel epigenetic factors, particularly miR-195, miR-15b, miR-21, and miR-29a, in driving a heightened angiogenic profile in TAT among elderly patients with cardiomyopathy compared to middle-aged patients. These miRNAs likely play a pivotal role in enhancing TAT’s neovascular potential with age. While further research is needed to elucidate the precise interactions between these miRNAs and co-expressed angiogenic factors in TAT, our results represent a significant stride in comprehending the intricate mechanisms underlying TAT’s remarkable capacity to produce angiogenic factors. This breakthrough holds great promise for advancing regenerative angiogenic therapies, particularly for individuals with ischemic cardiomyopathy.

## 6. Study Limitation

One of the limitations of this study is the small sample size, which could potentially restrict the generalizability of the findings. However, we believe that despite the challenge of recruiting such specific patients, we have managed to assemble a relatively homogeneous patient group, which may offset this limitation. Additionally, the fact that the only source of thymic fat is from ischemic cardiomyopathy subjects undergoing coronary artery bypass graft (CABG) surgery with cardiopulmonary bypass precludes the possibility of conducting this type of study using a control group of healthy subjects.

## Figures and Tables

**Table 1:** Anthropometric and biochemical characteristics of the patient groups.

Variables	Middle-Aged (*n* = 8)	Elderly (*n* = 10)	*p* Value
Age (years)	56.63 ± 1.72	74 ± 1.09	0.0001
Sex (M/W)	5/3	6/4	NS
BMI (kg/m^2^)	28.75 ± 1.74	29.40 ± 1.44	NS
Glucose (mg/dL)	104.12 ± 7.61	101.50 ± 5.33	NS
Triglycerides (mg/dL)	110.12 ± 10.36	116.80 ± 12.53	NS
Cholesterol (mg/dL)	129.00 ± 14.60	156.00 ± 10.06	NS

Participants (*n* = 18) were classified according to aging. Data are presented as the mean ± SEM. Comparisons between elderly and middle-aged groups were performed using the Student’s *t*-test, and *p* < 0.05 is considered statistically significant. Abbreviations: BMI = body mass index, NS = non-significant.

## Data Availability

All data generated or analyzed during this study are included in this article. Further enquiries can be directed to the corresponding author. Informed consent was obtained from all subjects involved in the study.

## Supplementary Materials

The following supporting information can be downloaded at: https://www.mdpi.com/article/10.3390/ijms241914456/s1.
