# Supplementary material for: A Network Comprised of miR-15b and miR-29a Is Involved in Vascular Endothelial Growth Factor Pathway Regulation in Thymus Adipose Tissue from Elderly Ischemic Cardiomyopathy Subjects
Source: Int J Mol Sci. 2023 Sep 22;24(19):14456. doi: 10.3390/ijms241914456 (PMC10572810; doi:10.3390/ijms241914456)
Supplement: Supplementary file 1 [file ijms-24-14456-s001.zip › ijms-2538542-supplementary.pdf]

SUPPLEMENTARY DATA

Table S1. miRNA predicted to regulate *VEGF-A*

Strong (44)	Weak (64)	Predicted (87)
hsa-miR-101-3p	hsa-miR-1252-3p	hsa-miR-17-5p
hsa-miR-106a-5p	hsa-miR-1277-5p	hsa-miR-20a-5p
hsa-miR-106b-5p	hsa-miR-1293	hsa-miR-93-5p
hsa-miR-107	hsa-miR-141-5p	hsa-miR-29b-2-5p
hsa-miR-125a-5p	hsa-miR-297	hsa-miR-197-5p
hsa-miR-126-3p	hsa-miR-299-3p	hsa-miR-150-5p
hsa-miR-133a-3p	hsa-miR-29c-3p	hsa-miR-34b-5p
hsa-miR-134-5p	hsa-miR-3163	hsa-miR-330-3p
hsa-miR-140-5p	hsa-miR-329-3p	hsa-miR-328-5p
hsa-miR-145-5p	hsa-miR-335-5p	hsa-miR-20b-5p
hsa-miR-147a	hsa-miR-362-3p	hsa-miR-520g-3p
hsa-miR-150-5p	hsa-miR-363-5p	hsa-miR-504-5p
hsa-miR-15a-5p	hsa-miR-3646	hsa-miR-638
hsa-miR-15b-5p	hsa-miR-3662	hsa-miR-874-5p
hsa-miR-16-5p	hsa-miR-369-3p	hsa-miR-541-5p
hsa-miR-17-5p	hsa-miR-374a-5p	hsa-miR-939-5p
hsa-miR-185-5p	hsa-miR-374b-3p	hsa-miR-1238-5p
hsa-miR-195-5p	hsa-miR-374b-5p	hsa-miR-1285-3p
hsa-miR-199a-5p	hsa-miR-3924	hsa-miR-1249-5p
hsa-miR-200b-3p	hsa-miR-3941	hsa-miR-548o-3p
hsa-miR-200c-3p	hsa-miR-410-3p	hsa-miR-1976
hsa-miR-203a-3p	hsa-miR-423-3p	hsa-miR-3122
hsa-miR-205-5p	hsa-miR-424-5p	hsa-miR-3132
hsa-miR-20a-5p	hsa-miR-4263	hsa-miR-3175
hsa-miR-20b-5p	hsa-miR-4483	hsa-miR-3184-3p
hsa-miR-21-5p	hsa-miR-4497	hsa-miR-4298
hsa-miR-29a-3p	hsa-miR-451b	hsa-miR-3650
hsa-miR-29b-3p	hsa-miR-4524a-5p	hsa-miR-3667-3p
hsa-miR-302d-3p	hsa-miR-4524b-5p	hsa-miR-3922-3p
hsa-miR-330-3p	hsa-miR-4719	hsa-miR-550b-2-5p
hsa-miR-34a-5p	hsa-miR-4735-5p	hsa-miR-4441
hsa-miR-34b-3p	hsa-miR-4789-5p	hsa-miR-4459
hsa-miR-361-5p	hsa-miR-495-3p	hsa-miR-4534
hsa-miR-372-3p	hsa-miR-497-5p	hsa-miR-3973
hsa-miR-373-3p	hsa-miR-5193	hsa-miR-4632-5p
hsa-miR-378a-3p	hsa-miR-567	hsa-miR-4644
hsa-miR-383-5p	hsa-miR-568	hsa-miR-4657
hsa-miR-503-5p	hsa-miR-5682	hsa-miR-4667-5p
hsa-miR-504-5p	hsa-miR-5688	hsa-miR-4685-5p
hsa-miR-520g-3p	hsa-miR-5692a	hsa-miR-4725-5p
hsa-miR-520h	hsa-miR-5692b	hsa-miR-4728-5p
hsa-miR-718	hsa-miR-5692c	hsa-miR-4730
hsa-miR-93-5p	hsa-miR-5694	hsa-miR-4739
hsa-miR-9-5p	hsa-miR-576-5p	hsa-miR-4743-3p
	hsa-miR-598-5p	hsa-miR-4746-3p

	hsa-miR-603	hsa-miR-4753-3p
	hsa-miR-646	hsa-miR-4756-5p
	hsa-miR-660-3p	hsa-miR-4769-5p
	hsa-miR-670-5p	hsa-miR-5002-5p
	hsa-miR-6745	hsa-miR-5189-5p
	hsa-miR-6748-5p	hsa-miR-5196-5p
	hsa-miR-6756-5p	hsa-miR-5591-5p
	hsa-miR-6759-5p	hsa-miR-5699-3p
	hsa-miR-6766-5p	hsa-miR-6072
	hsa-miR-6769a-5p	hsa-miR-6729-5p
	hsa-miR-6769b-5p	hsa-miR-6731-5p
	hsa-miR-6793-5p	hsa-miR-6753-5p
	hsa-miR-6838-5p	hsa-miR-6754-5p
	hsa-miR-6870-3p	hsa-miR-6774-5p
	hsa-miR-6871-3p	hsa-miR-6777-5p
	hsa-miR-7-1-3p	hsa-miR-6785-5p
	hsa-miR-7-2-3p	hsa-miR-6791-5p
	hsa-miR-8485	hsa-miR-6795-5p
	hsa-miR-95-5p	hsa-miR-6797-5p
		hsa-miR-6806-3p
		hsa-miR-6809-3p
		hsa-miR-6818-3p
		hsa-miR-6825-5p
		hsa-miR-6827-5p
		hsa-miR-6830-3p
		hsa-miR-6832-5p
		hsa-miR-6848-3p
		hsa-miR-6852-5p
		hsa-miR-6856-5p
		hsa-miR-6860
		hsa-miR-6873-3p
		hsa-miR-6875-3p
		hsa-miR-6876-5p
		hsa-miR-6879-3p
		hsa-miR-6887-5p
		hsa-miR-7107-3p
		hsa-miR-7112-5p
		hsa-miR-7156-5p
		hsa-miR-7160-3p
		hsa-miR-7851-3p
		hsa-miR-8085
		hsa-miR-8089

Table S2. TargetScan score calculation to miRNA-mRNA binding sites within *VEGF-A*

miRNA	Position	Seed match	Context++	Context++ (%)	Weight	Conservation	Pct
**hsa-miR-29a-3p**	1758-1765	8mer	-0.61	99	-0.46	4.186	0.89
**hsa-miR-29b-3p**	1758-1765	8mer	-0.61	99	-0.46	4.186	0.89
**hsa-miR-15b-5p**	292-299	8mer	-0.51	99	-0.51	3.981	0.82
**hsa-miR-16-5p**	292-299	8mer	-0.55	99	-0.55	3.981	0.82
**hsa-miR-195-5p**	292-299	8mer	-0.55	99	-0.55	3.981	0.82

Table S3. Gene aliases and names

GENE	NAME
**AKT2**	AKT serine/threonine kinase 2
**AKT3**	AKT serine/threonine kinase 3
**AXIN2**	Axin 2
**BIRC5**	Baculoviral IAP repeat containing 5
**FGF2**	Fibroblast growth factor 2
**FGFR1**	Fibroblast growth factor receptor 1
**FOS**	Proto-oncogene, AP-1 transcription factor subunit
**GSK3B**	Glycogen synthase kinase 3 beta
**JAG1**	Jagged canonical Notch ligand 1
**KDR**	Kinase insert domain receptor
**KRAS**	KRAS proto-oncogene, GTPase (KRAS)
**NOTCH2**	notch receptor 2
**PDGFA**	platelet derived growth factor subunit A
**PDGFB**	platelet derived growth factor subunit B
**PDGFC**	platelet derived growth factor C
**PDGFRA**	platelet derived growth factor receptor alpha
**PDGFRB**	platelet derived growth factor receptor beta
**PIK3CG**	phosphatidylinositol-4,5-bisphosphate 3-kinase catalytic subunit amma
**PIK3R1**	Homo sapiens phosphoinositide-3-kinase regulatory subunit 1
**RAF1**	Raf-1 proto-oncogene, serine/threonine kinase
**RASA1**	RAS p21 protein activator 1
**RHOB**	ras homolog family member B
**STAT3**	signal transducer and activator of transcription 3
**VEGFA**	vascular endothelial growth factor A
**WNT7A**	Wnt family member 7A

Table S4. Angiogenic pathway of differentially expressed target genes

PATHWAY	GENES
**Angiogenesis**	VEGFA,KDR, FGF2, PDGFRB, PIK3CG, PDGFRA, PDGFC, RHOB, JAG1, NOTCH2, WNT7A, FOS,
**Apoptosis signaling pathway (P00006)**	PIK3CG, FOS
**Hypoxia response via HIF activation (P00030)**	PIK3CG
**Cadherin signaling pathway (P00012)**	WNT7A
**Endothelin signaling pathway (P00019)**	PIK3CG
**FGF signaling pathway (P00021)**	PIK3CG, FGF2
**Hypoxia response via HIF activation (P00030)**	PIK3CG
**Notch signaling pathway (P00045)**	NOTCH2, JAG1

**PDGF signaling pathway (P00047)**	PIK3CG, FOS, RHOB, PDGFRA, PDGFRB
**VEGF signaling pathway (P00056)**	PIK3CG, KDR, VEGFA
**Wnt signaling pathway (P00057)**	WNT7A

Table S5. Gene expression primers

GENE	Ref.Seq	Product length	Fw primer sequence(5' ->3')	Rv primers sequence(5' ->3')
**AKT2**	NM_001243027.3	206	TGCCACCATGAATGAGGTGAATA	TGCAGGCAGCGTATGACAAA
**AKT3**	NM_001206729.2	704	TAATGGGGGCGAGCTGTTTT	AGTTACCCAGCATGCCACAA
**AXIN2**	NM_004655.4	182	TGGCAACTCAGTAACAGCCC	AGTTCCTCTCAGCAATCGGC
**BIRC5**	NM_001012270.2	382	ACGACCCCATGCAAAGGAAA	CTGGTAAGCCCGGGAATCAA
**FGF2**	NM_002006.5	121	TCCACCTATAATTGGTCAAAGTGGT	CATCAGTTACCAGCTCCCCC
**FGFR1**	NM_023110.3	175	ATTTCTGCCTTGGCCCTACC	CTAGCGCAGTCTTTGGGGAA
**FOS**	NM_005252.4	875	CAAGCGGAGACAGACCAACT	ACACACTCCATGCGTTTTGC
**GSK3B**	NM_002093.4	333	GGATTCGTCAGGAACAGGACA	TTAGCATCTGACGCTGCTGT
**JAG1**	NM_000214.3	883	GGCCGAGGTCCTATACGTTG	ACACAAGGTTTGGCCTCACA
**KDR**	NM_002253.4	123	CGGTCAACAAAGTCGGGAGA	CAGTGCACCACAAAGACACG
**NOTCH2**	NM_024408.4	73	CTACAGTTGTCGCTGCTTGC	GTTGGAGAGGCACTCGTTGA
**PDGFB**	NM_002608.4	573	GCCAGCGCCCATTTTTCAT	GAAAAGGAACACGGCAGTCG
**PDGFC**	NM_016205.3	411	CTGCCTCTTGTTTCCAATGCC	AAGCACAGGAAAAGGGTGCT
**PDGFRA**	NM_001347828.2	392	TGTGGGACATTCATTGCGGA	GCTCACTTCCAAGACCGTCA
**PDGFRB**	NM_002609.4	173	CAAGGACACCATGCGGCTTC	AGCAGGTCAGAACGAAGGTG
**PIK3CG**	NM_001282426.2	141	TGGATATGAAGGGAGCCCCA	CATGCCCTATGCGACCTGAT
**PIK3R1**	NM_181504.4	280	TGAGCTCAGCCAAGGAAACT	GTCCCGTCTGCTGTATCTCG
**RAF1**	NM_001354689.3	656	ATGAGCTTGCATGACTGCCT	CAAAATCGTCTGGACCACGC
**RASA1**	NM_022650.3	606	GGTTTCTGCTCAGCTCCCAT	TGGTGGTATTTGTAGGCCACT
**RHOB**	NM_004040.4	239	TCATGTGCTTCTCGGTGGAC	AGAGCACTCGAGGTAGTCGT
**STAT3**	NM_001369520.1	174	TGTGACACCATTCATTGATGCAG	GTAGGCGCCTCAGTCGTATC
**VEGFA**	NM_003376.6	442	CTTGCCTTGCTGCTCTACCT	GGGATTTCTTGCGCTTTCGT
**WNT7A**	NM_004625.4	505	ACTTAGGGGTAAGGAGGGGC	GCTGGACCCAAAGCAAAGTG

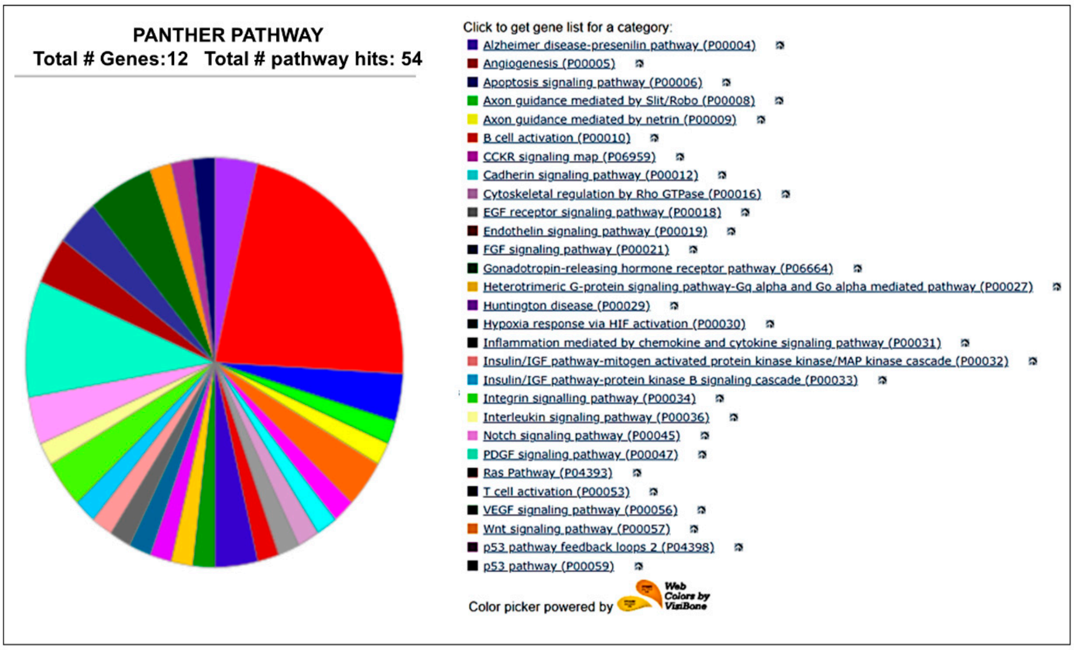
PANTHER PATHWAY
Total # Genes:12 Total # pathway hits: 54
Click to get gene list for a category:
Alzheimer disease-presenilin pathway (P00004)
Angiogenesis (P00005)
Apoptosis signaling pathway (P00006)
Axon guidance mediated by Slit/Robo (P00008)
Axon guidance mediated by netrin (P00009)
B cell activation (P00010)
CCKR signaling map (P06959)
Cadherin signaling pathway (P00012)
Cytoskeletal regulation by Rho GTPase (P00016)
EGF receptor signaling pathway (P00018)
Endothelin signaling pathway (P00019)
FGF signaling pathway (P00021)
Gonadotropin-releasing hormone receptor pathway (P06664)
Heterotrimeric G-protein signaling pathway-Gq alpha and Go alpha mediated pathway (P00027)
Huntington disease (P00029)
Hypoxia response via HIF activation (P00030)
Inflammation mediated by chemokine and cytokine signaling pathway (P00031)
Insulin/IGF pathway-mitogen activated protein kinase kinase/MAP kinase cascade (P00032)
Insulin/IGF pathway-protein kinase B signaling cascade (P00033)
Integrin signalling pathway (P00034)
Interleukin signaling pathway (P00036)
Notch signaling pathway (P00045)
PDGF signaling pathway (P00047)
Ras Pathway (P04393)
T cell activation (P00053)
VEGF signaling pathway (P00056)
Wnt signaling pathway (P00057)
p53 pathway feedback loops 2 (P04398)
p53 pathway (P00059)
Color picker powered by
Web Colors by VisiBone

Figure S1
